# Congenital cataract in children: Age at surgery and diagnosis-treatment interval in the Brazilian Unified Health System in Salvador (BA), Brazil

**DOI:** 10.1590/1984-0462/2026/44/2025337

**Published:** 2026-10-19

**Authors:** Igor Mello do Nascimento, Pollyanna Santos Suzart, Dayse Cury de Almeida Oliveira

**Affiliations:** a Escola Bahiana de Medicina e Saúde Pública, Salvador, Bahia, Brazil.; b Instituto Brasileiro Oftalmologia e Prevenção a Cegueira, Salvador, Bahia, Brazil.

**Keywords:** Cataract, Blindness, Neonatal screening, Amblyopia, Ophthalmology, Pediatrics, Catarata, Cegueira, Triagem neonatal, Ambliopia, Oftalmologia, Pediatria

## Abstract

**Objective:**

To determine the age at first congenital cataract surgery and the diagnosis-to-treatment interval at a specialized service in Salvador, Brazil, quantifying treatment delay and characterizing the clinical-epidemiological profile of the population.

**Methods:**

Retrospective, observational case series with an exploratory analytical component, including pediatric patients who underwent congenital cataract surgery through the Brazilian Unified Health System between 2015 and 2024. The primary outcomes were age at first surgery and the diagnosis-to-treatment interval; as a secondary, exploratory aim, factors associated with surgical age were examined. Student’s *t*-test, the Mann-Whitney U test, and linear regression were used, with estimates reported as coefficients and 95% confidence intervals - 95%CIs (p<0.05).

**Results:**

A total of 187 patients (274 eyes) were included, predominantly male (60.4%) and bilateral (54.5%). The median age at surgery was four years (interquartile range [IQR] 2.0-6.0), and no child was operated on within the recommended window; only 13 (7.0%) underwent surgery before one year of age. The diagnosis-to-treatment interval was adequate in 48.1% and delayed in 51.9%. In the exploratory adjusted model, no variable was independently associated with surgical age (adjusted R^2^=0.029). The median remained 4.0 years even in the absolute-surgical-indication subgroup, indicating that greater clinical severity did not translate into earlier surgery.

**Conclusions:**

Surgery occurred well beyond the recommended window. As the in-hospital interval was relatively efficient, the delay appears to lie upstream of referral, reinforcing the need to strengthen neonatal screening and early detection in primary care.

## RESUMO

**Objetivo:**

Determinar a idade na primeira cirurgia de catarata congênita e o intervalo entre diagnóstico e tratamento em um serviço especializado de Salvador, Bahia, quantificando o atraso e caracterizando o perfil clínico-epidemiológico da população.

**Métodos:**

Série de casos retrospectiva e observacional, com componente analítico exploratório, incluindo pacientes pediátricos submetidos à cirurgia de catarata congênita pelo Sistema Único de Saúde entre 2015 e 2024. Os desfechos primários foram a idade à primeira cirurgia e o intervalo diagnóstico-tratamento; como objetivo secundário, exploratório, examinaram-se fatores associados à idade cirúrgica. Empregaram-se os testes *t* de Student e Mann-Whitney e regressão linear, com estimativas em coeficientes e intervalo de confiança de 95% - IC95% (p<0,05).

**Resultados:**

Foram incluídos 187 pacientes (274 olhos), com predomínio de meninos (60,4%) e casos bilaterais (54,5%). A mediana de idade à cirurgia foi de quatro anos (intervalo interquartil [IIQ] 2,0-6,0), e nenhuma criança foi operada no período ideal; apenas 13 (7,0%) o foram antes de um ano. O intervalo diagnóstico-tratamento foi adequado em 48,1% e tardio em 51,9%. No modelo ajustado, exploratório, nenhuma variável foi independentemente associada à idade cirúrgica (R^2^ ajustado=0,029). A mediana permaneceu em 4,0 anos mesmo no subgrupo com indicação cirúrgica absoluta, demonstrando que a maior gravidade clínica não resultou em cirurgia mais precoce.

**Conclusões:**

A cirurgia ocorreu muito além do período recomendado. Como o intervalo intra-hospitalar foi relativamente eficiente, o atraso parece situar-se na etapa anterior ao encaminhamento, reforçando a necessidade de fortalecer a triagem neonatal e a detecção precoce na atenção primária.

## INTRODUCTION

Congenital cataract is the leading cause of reversible childhood blindness, with a prevalence of 1-6 cases per 10 thousand live births in developed countries and 5-15 per 10 thousand in developing regions.^1^ ^,^ ^2^ In Brazil, this pathology accounts for 12.8% of pediatric blindness.^3^ The lack of early intervention results in stimulus deprivation amblyopia and permanent visual impairment,^4^ causing an impact on the quality of life comparable to that of pediatric neoplasms or rheumatic diseases.^5^

The interval until surgical treatment is the main modifiable prognostic factor. Surgery, the treatment of choice in most cases, should be performed at four to six weeks of life for unilateral cases, and at six to eight weeks for bilateral cases. Procedures performed after these periods are associated with worse visual outcomes, even with appropriate management.^4^ ^,^ ^6^ ^,^ ^7^

Absolute surgical indications in pre-verbal patients include dense cataract with an obscured red reflex under mydriasis, strabismus with unilateral cataract, or nystagmus accompanied by bilateral cataract. In selected cases featuring incomplete opacities that spare the visual axis and allow clear visualization of the fundus, surgery may be postponed, and conservative management with amblyopia monitoring is permissible.^6^

Therefore, delays in diagnosis and referral constitute critical barriers. International protocols recommend the red reflex test (RRT) at birth and at six weeks of life, coinciding with the vaccination schedule. In settings where screening is routine, earlier diagnosis and timely treatment are observed.^8^ However, delayed diagnosis persists in various scenarios, associated with a lack of public awareness, deficient infrastructure, and insufficient professional training regarding the RRT.^9^ ^,^ ^10^ ^,^ ^11^ ^,^ ^12^

Accordingly, the primary aim of this study was to determine the age at first congenital cataract surgery and the diagnosis-to-treatment interval at a specialized service in Salvador (BA), Brazil, thereby quantifying the magnitude of any treatment delay and characterizing the clinical-epidemiological profile of the population. As a secondary, exploratory aim, we examined clinical and demographic factors potentially associated with age at surgery; no formal inferential hypothesis was prespecified, and the regression analyses are therefore hypothesis-generating rather than confirmatory.

## METHOD

This is a retrospective, observational case series with an exploratory analytical component conducted at Hospital Humberto Castro Lima (HHCL), an ophthalmology reference center in Salvador (BA) that provides services to the Unified Health System (SUS) and private health insurances. The study included a consecutive sample of pediatric patients (zero to ten years old, as defined by the World Health Organization - WHO) who underwent congenital cataract surgery via the Unified Health System (SUS) between January 1, 2015, and December 31, 2024, and whose medical records contained sufficient clinical data to fulfill the variables of interest. No a priori sample-size calculation was performed, characterizing a convenience sample.

Data collected included age at the first surgical approach and the diagnosis-treatment interval, alongside sociodemographic information (sex, place of residence). The collected clinical data encompassed cataract laterality, systemic comorbidities (e.g., genetic syndromes, hematological diseases, and congenital infections), and specific ophthalmic data of the operated eyes, such as cataract morphological type, presence of other ocular abnormalities, and preoperative visual acuity.

The primary outcomes were age at first surgery and the diagnosis-to-treatment interval; the analysis of factors associated with age at surgery constituted a secondary, exploratory objective without a prespecified inferential hypothesis.

Age at the first surgical approach was classified as early (<1 month), adequate (1-3 months), or delayed (>3 months). The diagnosis-treatment interval was defined as the time elapsed between the first confirmatory clinical record of pediatric cataract at the specialized service and the date of the first surgery. This interval, described in months, was considered delayed if it exceeded three months.^13^ ^,^ ^14^

It is acknowledged that, for referred patients, this date may not correspond to the initial detection of an altered red reflex in primary care or in their hometowns. Instead, it represents the moment of specialized diagnosis and the initiation of definitive surgical planning at the reference center.

Data were extracted from medical records, managed in Research Electronic Data Capture (REDCap), and analyzed using Microsoft Excel^®^ 2019 and SPSS 14.0. Continuous variables were summarized as mean and standard deviation or, when the distribution was non-normal, as median and interquartile range (IQR); categorical variables, as absolute and relative frequencies. Between-group comparisons used Student’s *t*-test or the Mann-Whitney U test, according to distribution.

The primary outcome, age at first surgery, was analyzed as a continuous variable. Because inclusion was conditional on surgery having occurred, no observations were censored and a time-to-event framework was not applicable; linear regression was therefore used. Each predictor was first assessed by simple (univariable) linear regression, after which all predictors were entered simultaneously into a multiple (multivariable) model (enter method), providing fully adjusted estimates irrespective of individual significance, without stepwise selection or interaction terms.

Predictors were selected on clinical grounds: sex, laterality, systemic comorbidities, patient origin, and absolute surgical indication. This last subgroup was defined a priori as patients whose most severely affected operated eye presented a total morphological cataract with severely compromised visual acuity (absence of fixation, hand motion, or counting fingers) and, in bilateral cases, accompanying strabismus or nystagmus; for these analyses, bilateral cases were classified by the eye of greater morphological severity or worse visual acuity.

Model adequacy was assessed by examining the standardized residuals. The normal P-P plot indicated that the residuals were normally distributed, the residual-versus-predicted scatterplot showed no evidence of heteroscedasticity, and the Durbin-Watson statistic (1.967) was consistent with independence of residuals. Minor departures from normality were compatible with the exploratory nature of the analysis. Multicollinearity in the multivariable model was assessed by the Variance Inflation Factor (range 1.012-1.048). Estimates are reported as unstandardized coefficients (β) with 95% confidence intervals (95%CI), with significance set at p<0.05.

## RESULTS

Between 2015 and 2024, 275 children underwent cataract surgery at the institution. After excluding 71 cases of acquired etiology (65 traumatic and six other) and 17 with incomplete records, the final sample comprised 187 children (274 operated eyes) with congenital cataract (Figure 1). The cohort was predominantly male (60.4%) and resident in the state capital (73.3%), with bilateral involvement in 54.5% and systemic comorbidities in 7.0% (Table 1).

**Figure 1.**
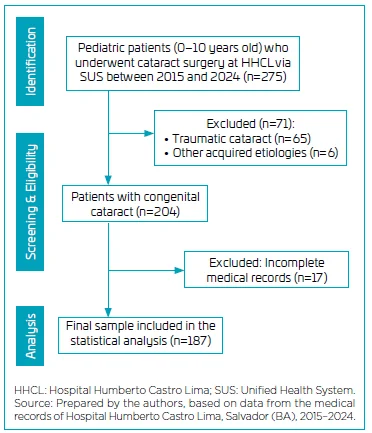
Strengthening the Reporting of Observational Studies in Epidemiology (STROBE) flow diagram of the study selection and enrollment process.

**Table 1.** Demographic and clinical data of the study sample (n=187).

Variable	n (%)
Sex	
Male	113 (60.4)
Place of residence	
State capital	137 (73.3)
Other cities	50 (26.7)
Comorbidities	
Yes	13 (7.0)
Laterality	
Unilateral	85 (45.5)
Bilateral	102 (54.5)

Source: Prepared by the authors, based on data from the medical records of Hospital Humberto Castro Lima, Salvador (BA), 2015-2024.

The associated systemic conditions were heterogeneous, each occurring in a single child: diabetes, epilepsy, low birth weight, galactosemia, homocystinuria, birth hypoxia, myelomeningocele, gestational rubella, gestational syphilis, and sickle cell trait; a further three patients (1.7%) had an unspecified syndromic condition.

Among the 274 evaluated eyes, most had no additional ocular abnormality (68.7%), strabismus being the most frequent associated finding (22.5%) (Table 2). Regarding morphological type, the most frequent forms were total (n=82), lamellar (n=66), posterior polar (n=42), and nuclear cataracts (n=34). Posterior subcapsular (n=23), mixed (n=11), anterior polar (n=8), sutural (n=4), and cortical cataracts (n=4) were also observed.

**Table 2.** Distribution of additional ophthalmological findings observed (n = 274 eyes).

Additional ophthalmological changes	n (%)
Strabismus	62 (22.5)
Persistence of the hyaloid artery	6 (2.2)
Nystagmus	4 (1.5)
Microspherophakia	4 (1.5)
Microphthalmia	2 (<1.0)
Lenticonus	2 (<1.0)
Subluxation of the lens	2 (<1.0)
Glaucoma	1 (<1.0)
Aniridia	1 (<1.0)
Microcornea	1 (<1.0)
No associated changes	188 (68.7)

Source: Prepared by the authors, based on data from the medical records of Hospital Humberto Castro Lima, Salvador (BA), 2015-2024.

Visual acuity, recorded in 199 of 274 eyes (72.6%), most often corresponded to “fixes and follows” (29.6%) and “counting fingers” (18.1%), with the remaining eyes distributed across categories ranging from absence of fixation and hand motion to 20/40 to 20/20.

The median age at the first procedure was four years (IQR 2.0-6.0), and no child was operated on within the recommended optimal window. A total of four children (2.1%) were operated on at ten years of age, the upper age limit for inclusion in the study. Only 13 patients (7.0%) underwent surgery before one year of age, all of whom were operated on between six and 12 months of age (Table 3). Of these, the majority were male (92.3%), from the capital (61.5%), and presented with bilateral cataracts (69.2%). None of the patients operated on during this period had associated systemic comorbidities.

**Table 3.** Sample distribution by patient age at the time of the first surgical procedure for cataract correction.

Patient age	Frequency	Percentage (%)	Cumulative percentage
Before 1 year of age (6 to 12 months)	13	7.0	7.0
At 1 year of age	23	12.3	19.3
At 2 years of age	21	11.2	30.5
At 3 years	28	15.0	45.5
At 4 years	28	15.0	60.4
At 5 years	18	9.6	70.1
At 6 years	14	7.5	77.5
At 7 years	14	7.5	85.0
At 8 years	13	7.0	92.0
At 9 years	11	5.9	97.9
At 10 years	4	2.1	100.0
Total	187	100.0	-

Source: Prepared by the authors, based on data from the medical records of Hospital Humberto Castro Lima, Salvador (BA), 2015-2024.

Age at surgery did not differ by sex or laterality (median four years in all groups; p=0.678 and p=0.987, respectively). Children from the capital were operated on later than those from the countryside (four years, IQR 2.5-7.0 vs. three years, IQR 1.0-5.0; p=0.031), while the difference associated with systemic comorbidities did not reach significance (five years, IQR 4.0-7.0 vs. four years, IQR 2.0-6.0; p=0.070).

Notably, the median age remained four years even in the prespecified absolute-surgical-indication subgroup (n=116), indicating that greater clinical severity was not accompanied by earlier surgery. Of the 116 patients in this subgroup, ten (8.6%) were operated on before one year of age, of which 90.0% were male, 80.0% had bilateral cases, and 100.0% had no systemic comorbidities. There was equivalence in origin, with patients evenly distributed between the capital and the countryside.

Consistent with this, the exploratory regression identified only countryside origin as associated with a lower age at surgery in the unadjusted analysis (β=-0.90; 95%CI -1.74 to -0.06; p=0.036); in the adjusted model no predictor remained independently associated (all p≥0.05), the model explained less than 3% of the variance (adjusted R² = 0.029), and no multicollinearity was detected (Table 4).

**Table 4.** Simple and multiple linear regression analysis for predictors of age at congenital cataract surgery.

Variables	Simple linear regression	p-value	Multiple linear regression	p-value
	β (95%CI)		β (95%CI)	
Absolute surgical indication (yes)	-0.73 (-1.50 to 0.03)	0.060	-0.78 (-1.57 to 0.01)	0.052
Systemic comorbidities (yes)	1.17 (-0.30 to 2.64)	0.117	1.28 (-0.20 to 2.76)	0.090
Patient origin (contryside)	-0.90 (-1.74 to -0.06)	0.036	-0.78 (-1.63 to 0.07)	0.070
Sex (female?)	0.04 (-0.73 to 0.81)	0.917	-0.03 (-0.79 to 0.74)	0.944
Laterality (bilateral?)	-0.06 (-0.81 to 0.70)	0.883	-0.07 (-0.83 to 0.69)	0.849

CI: confidence interval; β: Unstandardized regression coefficient.

Reference categories: capital (origin), male (sex), unilateral (laterality), and absence (comorbidities and absolute indication). Coefficients express the change in age at surgery, in years, relative to the reference category.

Source: Prepared by the authors, based on data from the medical records of Hospital Humberto Castro Lima, Salvador (BA), 2015-2024.

The interval between specialized diagnosis and surgery was adequate (≤3 months) in 90 patients (48.1%) and delayed in 97 (51.9%), with 9.1% waiting more than one year (Figure 2).

**Figure 2.**
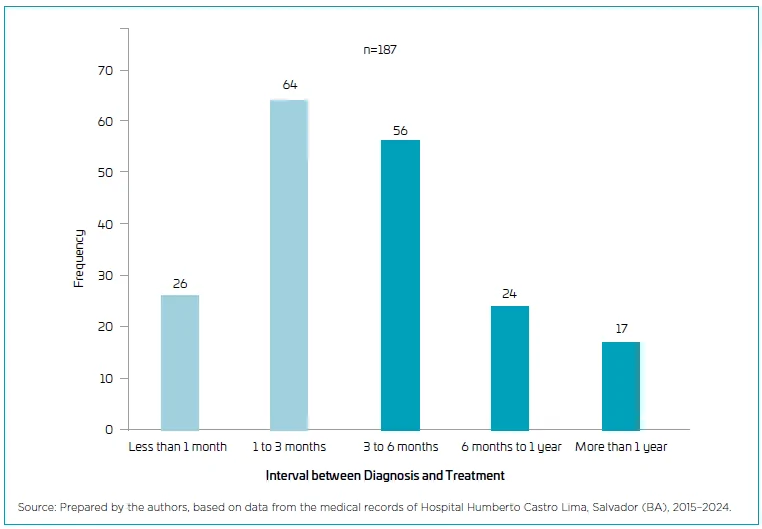
Distribution of the sample (n=187) according to the interval between diagnosis and surgical treatment. The dashed bars represent the “appropriate” interval (up to three months), and the solid gray bars represent the “late” interval (over three months).

## DISCUSSION

The median age of four years for the first surgical intervention considerably exceeds international recommendations, and this figure remained unchanged even within the absolute-surgical-indication subgroup, in which the amblyogenic risk is highest. This constitutes the central finding of the present study.

Preoperative functional assessment legitimately influences surgical timing: methods such as Teller acuity cards, Lea Hyvärinen (LEA) symbols, or HOTV optotypes - a chart restricted to the letters H, O, T and V - quantify the visual impact of the opacity,^15^ and because congenital cataracts do not invariably cause immediate severe impairment, structured monitoring can justify the safe postponement of surgery in selected partial cataracts.^4^

However, while preoperative functional assessment may justify a safe postponement in partial cataracts, the persistence of delayed intervention in cases with absolute indications in our sample suggests that barriers to access and failures within the health system are primary determinants of this late approach.

While surgery is generally performed before four months of age in high-income countries,^16^ our results approximate those of South and Central Asia, regions likewise marked by limited neonatal screening.^17^ ^,^ ^18^ In the Brazilian scenario, the median age of 48 months aligns with studies conducted in São Paulo,^19^ ^,^ ^20^ ^,^ ^21^ although it diverges from more recent national series that report averages close to eight months, highlighting regional disparities in access to specialized diagnosis.^22^

The epidemiological profile reinforces this regional reading. The male predominance (60.4%) corroborates part of the Brazilian literature,^23^ although not universal, since other national samples lack this prevalence;^22^ the sex ratio thus appears to vary with local demographics and referral flow. Consistently, sex was not an independent predictor of surgical age in our sample.

Children with systemic comorbidities were, on average, operated later, which may reflect the greater complexity of these patients, although this difference did not reach statistical significance. In many cases, severe systemic manifestations can hinder the initial perception of the cataract or lead to the prioritization of other clinical emergencies, thereby postponing the ocular diagnosis. Additionally, the need for rigorous medical stabilization prior to anesthesia may make postponement a conduct grounded in patient safety.^24^ Thus, a prolonged surgical interval may reflect both the appropriate management of global risks and a potential difficulty in identifying the disease in children with complex systemic conditions. This association should be regarded as hypothesis-generating rather than established.

Patient origin was associated with surgical age only in the unadjusted analysis, the association not persisting after adjustment. Although the literature consistently reports greater delays in rural populations,^10^ ^,^ ^17^ ^,^ ^18^ attributed in Brazil to the scarcity of specialized regional centers,^19^ this divergent finding must be read cautiously, as the underlying mechanisms were not measured.

Two non-exclusive explanations may be hypothesized but not demonstrated: an information bias, whereby families may register capital-based addresses and attenuate geographic differences; and a referral-selection effect, whereby patients arriving from outside the capital may already represent a pre-filtered subset.

Bilateral involvement predominated (54.5%), in line with the literature.^9^ ^,^ ^24^ ^,^ ^25^ ^,^ ^26^ Although laterality did not influence overall surgical age, a clinically relevant pattern emerged among the few children operated before one year of age, of whom 69.2% in the full sample and 80.0% within the absolute-indication subgroup had bilateral cataracts. This concentration of bilateral cases among the rare early procedures supports the hypothesis that unilateral cataracts are recognized later: the functional integrity of the fellow eye masks the deprivation and delays both familial perception and the search for specialized care.^17^ ^,^ ^18^ ^,^ ^24^

The exploratory regression identified no independent predictor of age at surgery. This absence of association is itself informative: even children meeting absolute surgical indications, those at greatest amblyogenic risk, were operated at the same median age (four years) as the overall sample. Clinical severity, therefore, did not translate into earlier surgery.

Notably, the adjusted R^2^ of 0.029 reveals that clinical and demographic variables explain less than 3% of the variance in surgical age. This indicates that the measured clinical and demographic profile accounts for only a negligible share of when surgery occurs. This pattern is consistent with the predominance of determinants external to the child’s clinical presentation, although such factors were not measured here and cannot be inferred from these data.

This interpretation is supported by the diagnosis-to-treatment interval. Although surgical timing as a whole was late, the interval between specialized diagnosis and surgery was adequate in almost half of the sample, with only 9.1% exceeding one year. This compares favorably with international studies in similar populations, which often report referral times longer than 12 months.^10^ ^,^ ^11^ ^,^ ^12^ ^,^ ^17^ The critical component of the delay therefore does not lie in the resolving capacity of the surgical unit, but in the therapeutic pathway preceding arrival at the reference center, pointing to primary detection and referral as the principal bottleneck.

The predominance of total cataracts is congruent with this hypothesis, as partial opacities tend to progress to complete forms when not treated early; among associated ocular findings, eye-movement disorders were the most frequent, consistent with prolonged visual deprivation.^4^

Finally, the initial procedure, whether or not it includes primary intraocular lens implantation, represents only the first step in a lifelong management that demands continuous follow-up, optical correction, and occlusion therapy to prevent amblyopia.^13^

This study has limitations inherent to its retrospective, single-center design. First, it is subject to selection bias: only children who underwent surgery at a tertiary reference center were analyzed, while those never referred, lost along the referral pathway, treated elsewhere, or managed conservatively for milder disease were not captured; the reported delays therefore characterize the operated population and may underestimate the true burden of delay.

Second, reliance on secondary records limited data completeness, notably preoperative visual acuity, which was often unrecorded given the difficulty of assessing pre-verbal patients, and this may have reduced the precision of the absolute-surgical-indication subgroup. Third, and most importantly, the design captures only the interval after specialized diagnosis: it cannot measure pre-tertiary delays, and variables such as primary-care screening, parental awareness, and referral waiting times remain unassessed. These constraints limit the generalizability of our findings but do not affect the central descriptive result.

Within the limits of a single-center case series, congenital cataract surgery in this SUS reference service occurred well beyond the recommended window, a robust and design-independent finding, even though the workflow after specialized diagnosis was comparatively efficient. Because the pre-referral interval could not be measured, the true total delay is likely greater still.

This pattern points to early detection and referral, rather than surgical capacity, as the main target for intervention. Reducing age at surgery will require strengthening neonatal screening and primary-care training to ensure equitable access, so that surgical timing reflects a reasoned clinical decision rather than a delayed diagnosis.

Future prospective studies tracking the complete patient journey, from parental recognition of symptoms to screening and referral times, will be essential to guide targeted public policy. In this regard, the exploratory analyses of the present study are best read as hypothesis-generating: by showing that the measured clinical and demographic variables explain almost none of the variation in surgical age, they redirect attention toward unmeasured external determinants, which future analytical designs should test directly.

## Data Availability

The database that originated the article is available with the corresponding author.
